# Cartilage contact pressure elevations in dysplastic hips: a chronic overload model

**DOI:** 10.1186/1749-799X-1-6

**Published:** 2006-10-03

**Authors:** Mary E Russell, Kiran H Shivanna, Nicole M Grosland, Douglas R Pedersen

**Affiliations:** 1 Department of Orthopaedics and Rehabilitation, University of Iowa, Iowa City, Iowa; 2 Department of Biomedical Engineering, University of Iowa, Iowa City, Iowa, USA

## Abstract

**Background:**

Developmental dysplasia of the hip (DDH) is a condition in which bone growth irregularities subject articular cartilage to higher mechanical stresses, increase susceptibility to subluxation, and elevate the risk of early osteoarthritis. Study objectives were to calculate three-dimensional cartilage contact stresses and to examine increases of accumulated pressure exposure over a gait cycle that may initiate the osteoarthritic process in the human hip, in the absence of trauma or surgical intervention.

**Methods:**

Patient-specific, non-linear, contact finite element models, constructed from computed tomography arthrograms using a custom-built meshing program, were subjected to normal gait cycle loads.

**Results:**

Peak contact pressures for dysplastic and asymptomatic hips ranged from 3.56 – 9.88 MPa. Spatially discriminatory cumulative contact pressures ranged from 2.45 – 6.62 MPa per gait cycle. Chronic over-pressure doses, for 2 million cycles per year over 20 years, ranged from 0.463 – 5.85 MPa-years using a 2-MPa damage threshold.

**Conclusion:**

There were significant differences between the normal control and the asymptomatic hips, and a trend towards significance between the asymptomatic and symptomatic hips of patients afflicted with developmental dysplasia of the hip. The magnitudes of peak cumulative contact pressure differed between apposed articular surfaces. Bone irregularities caused localized pressure elevations and an upward trend between chronic over-pressure exposure and increasing Severin classification.

## Background

In the absence of trauma or any surgical intervention, patients afflicted with developmental dysplasia of the hip (DDH) tend to develop osteoarthritis (OA) much earlier than the population norm. Over a lifetime, chronic accumulation of locally elevated cartilage stress exposure may mechanically trigger biologic pathways leading to OA.

As a major load-bearing joint, the hip must sustain more than three times body weight during normal human gait [1-3]. DDH is a condition in which abnormal juxtaposition of the developing femoral head and acetabulum leads to a shallow acetabulum, femoral anteversion, and/or capsular laxity; thus, destabilizing the hip and leaving it prone to elevated cartilage contact pressure and subluxation. Bone incongruities of DDH cause locally elevated contact pressures on articular cartilage, and when accumulated over many years, may lead to cartilage degeneration and osteoarthritis [4-6]. Subluxation invariably leads to degenerative joint disease[7,8].

Descriptive assessment schemes of degenerative hip disease are generally 4-bin groupings based on apparent radiographic morphology, from normal to bad; Severin[9] (DDH), Bucholtz-Ogden[10] (DDH), and Boyer[11] (Slipped Capital Femoral Epiphysis). Little is done to differentiate the wide range of intermediate conditions within each group; even the 'normal' asymptomatic hip in unilateral hip disease may be far from normal in terms of subtle incongruities giving rise to transient cartilage stress elevations during ordinary activities of daily living. The chronic stress tolerance of cartilage has not been extensively studied; however, it is acknowledged that spatially averaged stress exposures may be a valid prognostic tool for OA[12]. Hadley, et al., (1990) indicated that chronic elevated stress exposure was likely a more significant factor in the progression of dysplasia and osteoarthritis than acute excessive stress values.

The objective of this study was to build three-dimensional, patient-specific, non-linear, contact finite element (FE) models from computed tomography (CT) arthrograms to calculate continuous gait cycle articular cartilage contact stress exposure that may initiate OA in the human hip. From these three-dimensional contact stresses, the cumulative pressure exposure per gait cycle was calculated, for symptomatic and asymptomatic hips. Individual peak cumulative (per gait cycle) contact pressures were correlated with Severin classification.

## Methods

We have a unique opportunity to investigate the link between abnormal mechanical load and the earlier-than-normal development of osteoarthritis in a natural hip in the absence of any known trauma or surgical intervention. All patients who had been managed with closed reduction for developmental dysplasia of the hip at the University of Iowa Hospital and Clinics between 1940 and 1969 were identified[7,13]. Clinical and radiographic data were available for 119 patients. The average duration of follow-up in 1995 was thirty years (range, 15 to 53 years). All radiographs were evaluated with the use of the anatomical classification of Severin[9]. The anteroposterior radiographs were also analyzed with the use of a digitizer program[14], with regard to the acetabular angle of Sharp[15,16], the center-edge CE-angle of Wiberg [17-19], and ten other common measurements. Intermediate reports have documented the progress of these patients[7,8,20-25]. Nineteen patients consented to CT arthrograms in addition to their standard clinical follow-up. The arthrogram protocol involved injection of a radio-opaque contrast agent into the joint space, which adhered to cartilage surfaces. Then the cartilage was visible on CT images as the dark region between the white subchondral bone and the bright contrast-filled space between the articulating surfaces. Radiological archives and clinical data were used in accordance with our institution's guidelines for human subjects research.

A total of six dysplastic and five asymptomatic hips (six patients) were modeled (Table 1). The average age of these patients at closed reduction of DDH hips was 1.25 years old, and the average age at the time of CT arthrogram acquisition was 40 years old. An age-matched normal hip model was generated from the 38 year old male subject of the Visible Human Project^® ^[26,27]. For dysplastic hips the average Wiberg center-edge angle was 24° (range 14°–34°) and, the average Sharp's angle was 42° (range 36°–49°). The asymptomatic hips' average Wiberg center-edge and Sharp's angles were 33° (range 26°–37°) and 38° (range 29°–44°), respectively.

**Table 1:** Patient Demographics

Patient	Sex	Weight (kg)	Age (years)	Side	Affected	CE angle	Sharp's angles	Severin score
Control	M	90	38	R	N	n/a	n/a	n/a

1	M	74	39	L	N	36.70	29.34	I
				R	**Y**	**21.87**	**42.90**	**I**
2	F	52	49	L	**Y**	**33.81**	**36.26**	**I**
				R	**Y**	**24.80**	**40.40**	**I**
3	F	63	32	R	N	28.54	35.88	I
4	F	51	53	L	**Y**	**25.20**	**38.85**	**II**
				R	N	26.06	37.97	I
5	F	65	28	L	N	32.94	39.94	I
				R	**Y**	**27.83**	**39.20**	**II**
6	F	61	42	L	**Y**	**14.17**	**49.48**	**III**
				R	N	32.56	43.75	I

These patients were selected to address three issues: **1) **Normal-appearing asymptomatic hips experience near-normal articular cartilage loading; **2) **Bone irregularities in incongruous dysplastic hips significantly elevate local articular cartilage stresses; **3) **Significant variability exists in chronic cumulative stresses of hips scaled as normal (Severin I).

**Figure 1:**
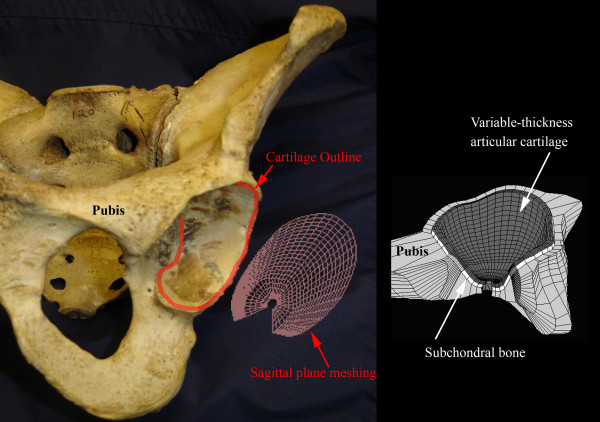
**Acetabular modeling**. The region of cartilage coverage is traced and projected onto a sagittal plane, where a 2D quadrilateral mesh is generated. The regular 2D mesh is projected onto the surface of the acetabulum.

Hip contact pressure distributions from DDH patient models were compared to the 'normal standard' of an age-matched hip from the Visible Human project[27]. Each FE model was developed from the axial planes of a 3-D CT volume. Features of interest for both the femoral head and the acetabulum were manually traced using BRAINS2 [28-30]. Commercially available meshing software produced inconsistent element aspect ratios in these highly irregular acetabular concavities. Therefore, the horseshoe shaped cartilage coverage region on the acetabulum was delineated for preferential meshing. Anatomical data were input to a custom-written in-house mesh-generating program[31,32] with the ability to maintain bone irregularities underlying variable thickness, smooth surface articular cartilage. The region of cartilage coverage was delineated and projected onto the sagittal plane, where a regular two-dimensional quadrilateral mesh was generated (Figure 1). The 2D mesh was projected back into the irregular 3D acetabular concavity, and expanded into the acetabular fossa for bone surface definition[33].

Cartilage geometries required additional considerations. For example, cartilage of uniform-thickness, generated by simply extruding the bone surface, included all the irregularities of the bone surface. Cartilage surfaces generated from direct outlines of the radio-opaque contrast enhanced cartilage surfaces in CT arthrograms included the flat spots of hip contact areas. These situations were addressed by smoothing the articular cartilage surface toward an ellipsoidal curvature, for both the acetabulum and the femoral head. The resulting variable thickness of cartilage in subsequent patient-specific FE models (0.5 to 2.8 mm, average of 1.8 mm) was similar to articular cartilage thicknesses observed in the CT slices[33,34].

Individual patient-specific finite element models were meshed with roughly 5,000 and 20,000 hexahedral elements assigned to the acetabulum and femoral head, respectively, and solved using ABAQUS (V6.4). The 3-D FE contact models were non-linear, deformable body, large-displacement and frictionless. The irregular femoral head surface was covered with three layers of smooth-surface, variable thickness cartilage (Young's Modulus, E = 12 MPa; Poisson's ratio, ν = 0.42). The 1-mm thick subchondral bone (E = 2 GPa, ν = 0.3) of the acetabular dome was supported by cancellous bone (E = 120 MPa, ν = 0.3) with a fixed boundary at the medial pelvic wall (Figure 1). This non-rigid pelvic bone support of the acetabulum exhibited natural compliance that facilitated attainment of continuous horseshoe shaped contact of a normal hip articulation[6,35,36].

**Figure 2:**
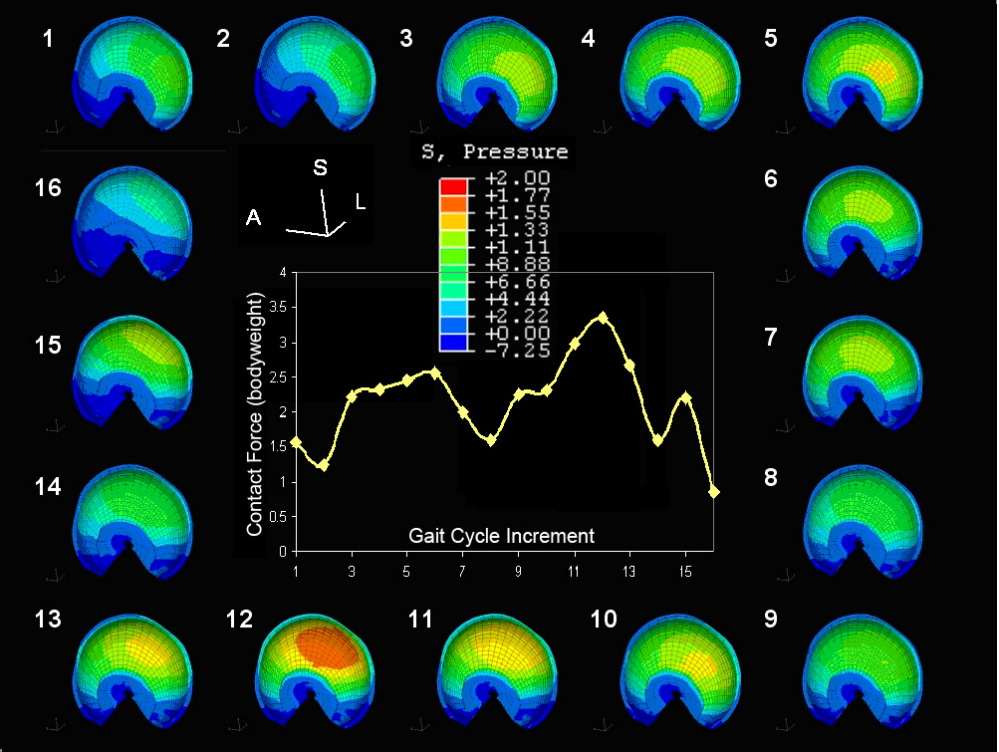
**Applied loads and normal hip contact contours**. Finite element control hip contact pressure contours at each gait cycle increment develop from the resultant contact force (inset) applied during gait stance phase kinematics.

Gait cycle kinematics and kinetics from the weight-bearing stance phase of a 67-kg 72-year-old man with a telemetered femoral component total hip reconstruction[1,3] were discretized into sixteen intervals from heel-strike through toe-off[37]. Femoral kinematics and joint contact force were expressed with respect to the pelvic reference frame. Loading and displacement were applied through a reference node whose location coincided with the computed center of the femoral head. The six degrees of freedom were specified as 3 rotations about and 3 loads along the three anatomical axes. The cited peak resultant loads (Figure 2 inset) were scaled by each individual's bodyweight.

As mesh refinement of the Visible Human male 'normal hip' model was increased, random centers of contact during the stance phase coalesced into a broad continuous horseshoe shaped contact area. The size of the elements on the two contacting surfaces was progressively decreased and peak contact pressure obtained was used for convergence. The normal hip model solution, which converged with 25,000 hexahedral elements, established the reference for DDH patients' hip model load distributions (Figure 2).

The spatial and temporal involvements of the acetabular and femoral articular cartilage were explicitly quantified from the pressure stress, P, at the centroid of every surface element at each of the 16 increments in the gait cycle. ABAQUS reports the contact pressure CPRESS and the contact area CAREA on the master surface. The apposing contact surface stresses were extrapolated from elements (S. Pressure): P=13∑i=13σii
 MathType@MTEF@5@5@+=feaafiart1ev1aaatCvAUfKttLearuWrP9MDH5MBPbIqV92AaeXatLxBI9gBaebbnrfifHhDYfgasaacH8akY=wiFfYdH8Gipec8Eeeu0xXdbba9frFj0=OqFfea0dXdd9vqai=hGuQ8kuc9pgc9s8qqaq=dirpe0xb9q8qiLsFr0=vr0=vr0dc8meaabaqaciaacaGaaeqabaqabeGadaaakeaacqqGqbaucqGH9aqpdaWcaaqaaiabigdaXaqaaiabiodaZaaadaaeWbqaaiabeo8aZnaaBaaaleaacqWGPbqAcqWGPbqAaeqaaaqaaiabdMgaPjabg2da9iabigdaXaqaaiabiodaZaqdcqGHris5aaaa@3BEE@, where σ_ii _is each of the three principal stresses. Elemental stress values greater than 0.3 MPa were identified. The sum of the respective element face areas was consistent with the ABAQUS reported contact area variable CAREA.

The per-cycle cumulative contact pressure, P_cumulative_, for an element was

Pcumulative=∑i=116(pi⋅Δti)
 MathType@MTEF@5@5@+=feaafiart1ev1aaatCvAUfKttLearuWrP9MDH5MBPbIqV92AaeXatLxBI9gBaebbnrfifHhDYfgasaacH8akY=wiFfYdH8Gipec8Eeeu0xXdbba9frFj0=OqFfea0dXdd9vqai=hGuQ8kuc9pgc9s8qqaq=dirpe0xb9q8qiLsFr0=vr0=vr0dc8meaabaqaciaacaGaaeqabaqabeGadaaakeaacqqGqbaudaWgaaWcbaGaee4yamMaeeyDauNaeeyBa0MaeeyDauNaeeiBaWMaeeyyaeMaeeiDaqNaeeyAaKMaeeODayNaeeyzaugabeaakiabg2da9maaqahabaWaaeWaceaacqWGWbaCdaWgaaWcbaGaemyAaKgabeaakiabgwSixlabfs5aejabdsha0naaBaaaleaacqWGPbqAaeqaaaGccaGLOaGaayzkaaaaleaacqWGPbqAcqGH9aqpcqaIXaqmaeaacqaIXaqmcqaI2aGna0GaeyyeIuoaaaa@4F87@, where *p*_*i *_was the pressure on an element during a gait cycle interval, and Δ*t*_*i *_was the fraction of time for that increment, (e.g., 1/16^th ^of a second for this gait cycle). The distributions of cumulative pressure (MPa-seconds per cycle) versus contact area (mm^2^) were also recorded. These new spatially discriminatory measures separate femoral from acetabular articular cartilage exposure locations and magnitudes with respect to the individual bones. To focus on the effects of patient-specific geometries, we assumed a common activity level of two million gait cycles per year, consistent with reports from pedometer monitored patients[38]. The cumulative pressure in MPa-seconds per gait cycle then incorporated typical patient activity level with three-dimensional mapping of focal pressure elevations due to bone irregularities passing through the contact area.

A chronic exposure damage threshold of 2-MPa was chosen for this study. The normal control had no known musculoskeletal or hip problems and, at 1.229 MPa-sec/cycle on the femoral head and 1.145 on the acetabulum (Figure 2), no spatially discriminatory cumulative pressure exceeded this 2 MPa damage threshold. Chronic over-pressure, P_chronic_, was calculated as

Pchronic=∑i=116((Pi−Pd)∗Δti)∗(2∗106cyclesyear)∗(year3.16∗107sec⁡)∗N
 MathType@MTEF@5@5@+=feaafiart1ev1aaatCvAUfKttLearuWrP9MDH5MBPbIqV92AaeXatLxBI9gBaebbnrfifHhDYfgasaacH8akY=wiFfYdH8Gipec8Eeeu0xXdbba9frFj0=OqFfea0dXdd9vqai=hGuQ8kuc9pgc9s8qqaq=dirpe0xb9q8qiLsFr0=vr0=vr0dc8meaabaqaciaacaGaaeqabaqabeGadaaakeaacqqGqbaudaWgaaWcbaGaee4yamMaeeiAaGMaeeOCaiNaee4Ba8MaeeOBa4MaeeyAaKMaee4yamgabeaakiabg2da9maaqahabaGaeiikaGIaeiikaGIaemiuaa1aaSbaaSqaaiabdMgaPbqabaGccqGHsislcqWGqbaudaWgaaWcbaGaemizaqgabeaakiabcMcaPiabgEHiQiabfs5aejabdsha0naaBaaaleaacqWGPbqAaeqaaOGaeiykaKcaleaacqWGPbqAcqGH9aqpcqaIXaqmaeaacqaIXaqmcqaI2aGna0GaeyyeIuoakiabgEHiQiabcIcaOmaalaaabaGaeGOmaiJaey4fIOIaeGymaeJaeGimaaZaaWbaaSqabeaacqaI2aGnaaGccqqGJbWycqqG5bqEcqqGJbWycqqGSbaBcqqGLbqzcqqGZbWCaeaacqqG5bqEcqqGLbqzcqqGHbqycqqGYbGCaaGaeiykaKIaey4fIOIaeiikaGYaaSaaaeaacqqG5bqEcqqGLbqzcqqGHbqycqqGYbGCaeaacqaIZaWmcqGGUaGlcqaIXaqmcqaI2aGncqGHxiIkcqaIXaqmcqaIWaamdaahaaWcbeqaaiabiEda3aaakiGbcohaZjabcwgaLjabcogaJbaacqGGPaqkcqGHxiIkcqqGobGtaaa@7A87@

where *P*_*i *_was the pressure for an element at a specific step in the gait cycle; *P*_*d *_was the pressure damage threshold above which cartilage damage occurs (*P*_*i *_- *P*_*d *_≥ 0); Δ*t*_*i *_was the fraction of time that the pressure *P*_*i *_was acting on the element; N was the number of years for which the chronic over-pressure was being calculated. The digitized measurements on 20-, 30- and 40-year follow-up films indicated no major joint morphology changes with the radiographs taken at the time of CT examination (< 3% for all parameters). Therefore, to maintain focus on the effects of patient-specific geometries on chronic cartilage loading, we assumed each model was a reasonable representation of that patient's hip for twenty years from skeletal maturity, and N was set to 20 years. Any contact pressure under the chronic exposure damage threshold was excluded as normal wear and tear (P_chronic _= 0).

Independent t-tests were performed to compare the normal control and the asymptomatic hips. Paired t-tests measured the significance of differences between two different populations: the asymptomatic and symptomatic hips, and between the acetabulae and femoral heads of each patient. Statistical parameters included the peak pressure, the peak cumulative pressure, the peak over-pressure dose, and the mean contact area.

## Results

### Cartilage contact pressure elevations

**Figure 3:**
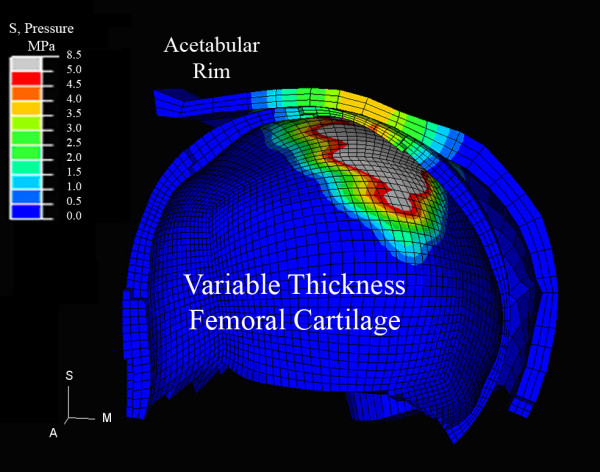
**Dysplastic hip cross-section**. A coronal section through finite element contact pressure contours at midstance is displayed on the isolated femoral head and acetabular articular cartilage. Note thin cartilage over a femoral 'bump' and localized peak pressures of 8.5 MPa associated with bone irregularities present within the incongruous DDH contact area. Differences in element size between the femoral head and the coarser acetabular mesh are accommodations for ABAQUS master-slave contact solutions.

A coronal cross-section through a patient's dysplastic hip (Figure 3) highlighted the variable cartilage thickness incorporated in the patient-specific modeling and also confirmed the assumption that bone abnormalities on the femoral head create localized supranormal pressures as random 'bumps' make contact with the acetabulum. The peak pressure magnitudes and the peak pressure timings within the gait cycle (increment number) were reported for the Visible Human control and for the 11 patient hips (Table 2). Separate acetabular and femoral head data also included the maximum accumulated pressure and the maximum chronic over-pressure exposure, along with Severin classification.

**Table 2:** Contact pressure data from age-matched control and all DDH patients' hips*

	Control	Patient 1		Patient 2		Patient 3	Patient 4		Patient 5		Patient 6	
	Right	Left	Right	Left	Right	Right	Left	Right	Left	Right	Left	Right
	Normal	asym	**DDH**	**DDH**	**DDH**	asym	**DDH**	asym	asym	**DDH**	**DDH**	asym
	
Severin Classification	I	I	**I**	**I**	**I**	I	**II**	I	I	**II**	**III**	I
**Acetabulum**												
Increment	12	12	**5**	**12**	**12**	12	**12**	6	12	**5**	**12**	12
Max pressure (MPa)	1.754	4.945	**6.984**	**3.563**	**8.298**	4.922	**5.25**	4.526	7.05	**6.87**	**8.587**	4.83
Max cum press (MPa-sec/cycle)	1.145	3.192	**4.686**	**2.447**	**4.699**	3.108	**3.835**	3.463	5.48	**5.44**	**6.553**	3.107
Max chronic press (MPa-years)	0	1.926	**3.399**	**0.566**	**3.416**	1.403	**2.323**	1.852	4.41	**4.36**	**5.764**	1.402

**Femoral Head**												
Increment	12	12	**4**	**12**	**12**	9	**12**	3	12	**5**	**12**	12
Max pressure (MPa)	1.89	6.462	**7.758**	**3.582**	**9.58**	6.096	**6.345**	5.15	7.38	**9.88**	**9.091**	5.48
Max cum press (MPa-sec/cycle)	1.229	4.702	**5.323**	**3.366**	**6.604**	3.815	**3.965**	3.827	5.43	**6.62**	**6.437**	3.45
Max chronic press (MPa-years)	0	3.419	**4.206**	**0.463**	**5.828**	2.298	**2.487**	2.313	4.34	**5.85**	**5.617**	1.829

*DDH in bold text cells, and asym is asymptomatic contralateral hip

### Chronic overload

**Figure 4:**
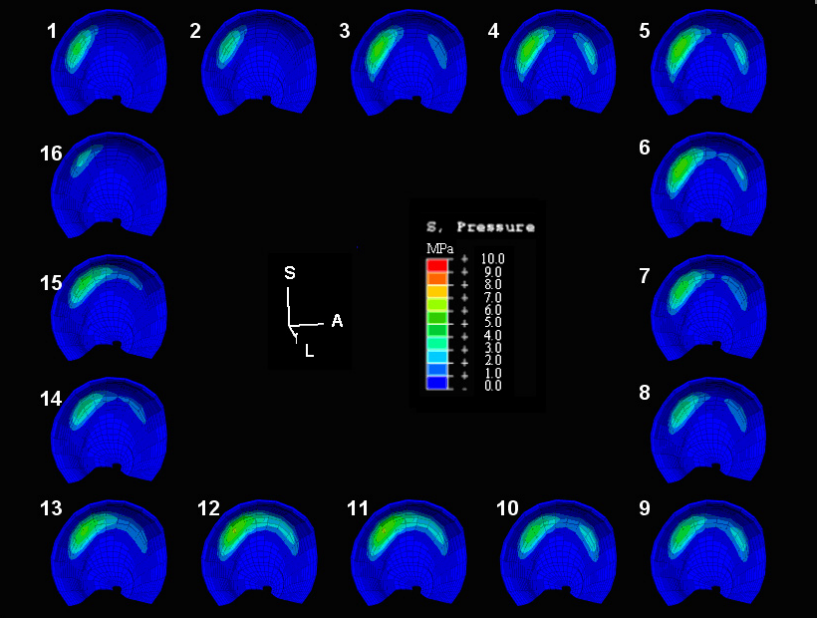
**Asymptomatic hip contact contours**. Gait cycle contact pressures on the asymptomatic acetabulum of patient 5 (Severin Grade I).

**Figure 5:**
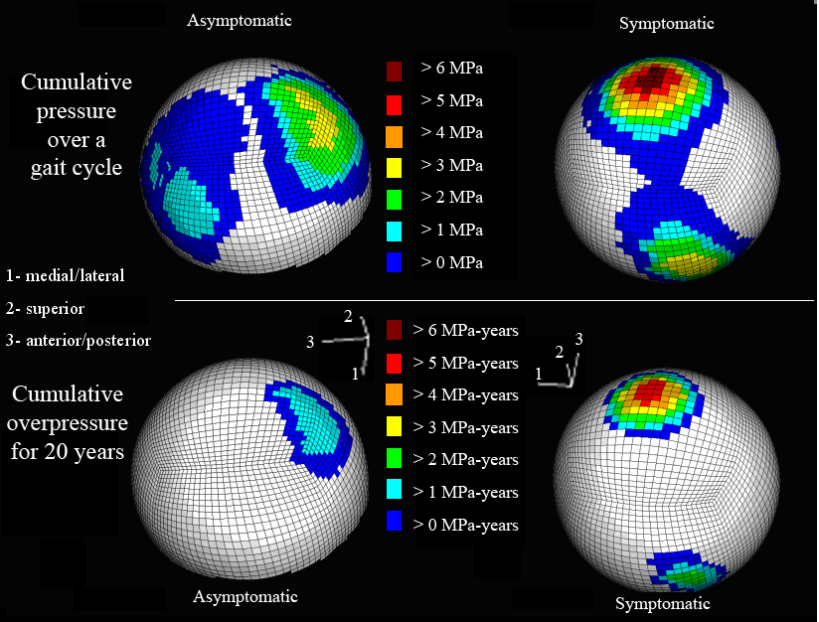
**Spatial distribution of cumulative femoral contact pressure**. (Top) Locations of femoral contact elements of Patient 6 experiencing cumulative pressure over the gait cycle and (bottom) overpressure exposure (> 2-MPa damage threshold) over 20 years.

A chronic exposure damage threshold of 2-MPa was chosen for this study. The cumulative stress per gait cycle and the chronic contact pressure spatial distribution results for patient 6 with a Severin III dysplastic hip and a Severin I asymptomatic hip were detailed for the acetabulum (Figure 4) and the femoral head (Figure 5). The contact pressure results from 11 hips are summarized in Table 2, alongside the normal control hip. Increment indicates the point in the 16-interval gait cycle stance phase at which the maximum contact pressure occurred. Statistical comparisons of the Table 2 results are presented in Table 3.

**Table 3:** Summary statistics between age-matched control and all DDH patients' hips

**Peak Pressure (MPa)**	**P-value**	**Asymptomatic Mean ± S.D.**	**DDH Mean ± S.D.**	**Normal**
Acetabulum		5.25 ± 1.02	6.59 ± 1.90	1.75
Normal v. Asymptomatic	0.0339			
Asymptomatic v Symptomatic	0.0729			
Femoral Head		6.11 ± 0.87	7.71 ± 2.41	1.89
Normal v. Asymptomatic	0.0116			
Asymptomatic v Symptomatic	0.0302			
Acetabulum v. Femoral Head	0.0021			

**Cumulative Pressure (MPa)**				

Acetabulum		3.67 ± 1.02	4.61 ± 1.40	1.15
Normal v. Asymptomatic	0.0761			
Asymptomatic v Symptomatic	0.0756			
Femoral Head		4.24 ± 0.81	5.39 ± 1.43	1.23
Normal v. Asymptomatic	0.0268			
Asymptomatic v Symptomatic	0.0762			
Acetabulum v. Femoral Head	0.0075			

**Chronic Overpressure (MPa-sec/cycle)**				

Acetabulum		2.20 ± 1.26	3.30 ± 1.77	0
Normal v. Asymptomatic	0.1865			
Asymptomatic v Symptomatic	0.0402			
Femoral Head		2.84 ± 1.02	4.08 ± 2.20	0
Normal v. Asymptomatic	0.0642			
Asymptomatic v Symptomatic	0.0758			
Acetabulum v. Femoral Head	0.0157			

**Contact Area (mm^**2**^)**				

Acetabulum		663 ± 55	569 ± 97	2233
Normal v. Asymptomatic	0.0008			
Asymptomatic v Symptomatic	0.2482			
Femoral Head		621 ± 53	614 ± 183	2265
Normal v. Asymptomatic	0.0023			
Asymptomatic v Symptomatic	0.7537			
Acetabulum v. Femoral Head	0.5993			

## Discussion

Normal hip contact results for the Visible Human male were trending towards significance or significantly different from the asymptomatic patient hips in all calculations (peak pressure, peak cumulative pressure, peak over-pressure exposure, and mean contact area). This corroborates the previous assertions that the contralateral hip in DDH is also altered by the condition[39]. Therefore, it seems reasonable to model the disease progression of OA against the age-matched normal control of the Visible Human male who experienced no known musculoskeletal disease or trauma in the hip.

There was a significant reduction in contact area from the Visible Human control to the asymptomatic acetabulae and femoral heads. Overall calculated contact areas, ranging from toe-off of a dysplastic patient (215 mm^2^) through the midstance of the normal control (2265 mm^2^), are consistent with literature values of 759 to 2317 mm^2 ^at midstance[4,5,40]. Normal hip contact centered in the postero-medial acetabular roof (Figure 2) supports previous reports of location and extent of contact during gait[3,6,36,41,42]. Dysplastic hip contact shifted toward the postero-superior rim. To a lesser degree, asymptomatic hip contact also shifted laterally (Figure 4), which is consistent with Mavcic's observations[43].

These three-dimensional, patient-specific model solutions incorporate continuous gait-cycle kinematics and kinetics, for which comparable published models do not exist. Therefore, results comparisons are limited to a composite of reported quasi-static solutions at selected positions. The range of acetabular and femoral articular cartilage contact pressures (1.75–8.59 MPa and 1.89–9.88 MPa, respectively) lie within the range of reported values, from 1–10 MPa in normal and dysplastic hips[44,45]. Peak contact pressures for dysplastic and asymptomatic hips ranged from 3.56 to 9.88 MPa. The timing of the peak pressure did not always coincide with the peak load during the midstance of the gait cycle (Table 2). This is a direct consequence of bone irregularities entering the contact area. Pressure magnitude is mainly dependent on the area available for contact. Due to the incongruous surfaces of the dysplastic hip, the changing contact area involvement and the concomitant peak contact pressure do not always coincide with the peak load.

Studies have shown that chronic transient pressure elevations on articular cartilage correlate closely with the progression and onset of OA over many years[4,12]. While those studies extrapolated cartilage loading from clinical antero-posterior planar radiographs, the current study utilized the patients' CT arthrograms as a full 3-dimensional window into the cumulative contact pressure distributions on cartilage[34]. For the Severin III hip of patient 6 (Table 2, Figure 5), the maximum cumulative pressure was 6.55 MPa-per-cycle, and 5.76 MPa-years over 20-years using a 2-MPa damage threshold. The asymptomatic hip of that patient had maximum cumulative gait pressures and over-pressure doses of 3.11 MPa-per-cycle and 1.83 MPa-years.

Spatially discriminatory cumulative contact pressures ranged from 2.45 to 6.62 MPa-per-gait-cycle. Chronic over-pressure doses, for 2 million cycles per year over 20 years, ranged from 0.463 to 5.85 MPa-years using a 2-MPa damage threshold. The spatial distributions of elements experiencing cumulative pressures are located within the superior and posterior portions of the acetabulum (Figure 4). The magnitudes of peak cumulative contact pressure differed between apposed articular surfaces, where bone irregularities cause localized pressure elevations (Figure 5), and there is an upward trend between the chronic over-pressure exposure and increasing Severin classification.

These FE models include the limiting assumptions of a common normal gait pattern and frictionless articulation. The friction coefficient of synovial joints is very low, and there is no available direct assessment of patient differences. While it is recognized that patient-specific gait does play a role in actual joint contact, we assume common gait kinematics to maintain focus on the effects of patient specific geometries on chronic cartilage loading. These models have shown that asymptomatic hips, which may be normal-appearing on plane radiographs, cannot be assumed to experience the near-normal articular cartilage loading: Results for the Visible Human male are trending towards significance or significantly different from the asymptomatic patient hips in all calculations. Significant variability exists in the chronic cumulative stresses of hips scaled as normal (Severin I). A dysplastic acetabulum has the lowest accumulated stress per gait cycle (2.45 MPa-sec/cycle) while an asymptomatic acetabulae records the highest accumulated stress of 5.48 MPa-sec. Dysplastic femoral heads have the lowest (3.37) and highest (6.6 MPa-sec/cycle) cumulative stresses.

Bone morphologic changes characteristic of a DDH patient population with a propensity to OA, act as the focal stress concentrators that may be the forerunner in cartilage degeneration. As shown, the femoral head cartilage is much thinner over bone surface irregularities and experiences increased pressure elevations when in contact with its acetabular counterpart. The FE models are realistic variable cartilage thickness 3-dimensional models with patient-specific anatomies derived from CT scans and incorporating pelvic thickness subchondral and cancellous bone to provide a compliant support of the acetabulum. The non-linear, large-displacement FE models, incorporating continuous gait cycle kinematics and kinetics, are able to discriminate not only local pressure elevations spatially, but also discriminate differences between contact pressure elevations on the acetabulum and the femoral head.

## Conclusion

There are significant differences between the normal hip and the asymptomatic hips and a trend towards significance between the asymptomatic and symptomatic hips of DDH patients. The magnitudes of peak cumulative contact pressure differ between apposed articular surfaces. Bone irregularities cause localized pressure elevations and an upward trend between chronic over-pressure exposure and increasing Severin classification.

## Competing interests

The author(s) declare that they have no competing interests.

## Authors' contributions

MER carried out the finite element analysis. KHS developed the code used to generate the finite element models. NMG oversaw the model analysis and development of the code. DRP conceived the study and participated in its design and coordination. All authors participated in writing the manuscript. All authors read and approved the final manuscript.
